# Base-excess chloride; the best approach to evaluate the effect of chloride on the acid-base status: A retrospective study

**DOI:** 10.1371/journal.pone.0250274

**Published:** 2021-04-29

**Authors:** Bulent Gucyetmez, Filiz Tuzuner, Hakan Korkut Atalan, Uğur Sezerman, Kaan Gucyetmez, Lutfi Telci

**Affiliations:** 1 Department of Anaesthesiology and Reanimation, Acıbadem Mehmet Ali Aydınlar University School of Medicine, Istanbul, Turkey; 2 General Intensive Care Unit, Acıbadem International Hospital, Istanbul, Turkey; 3 General Intensive Care Unit, Acıbadem Taksim Hospital, Istanbul, Turkey; 4 Department of Anaesthesiology, Ataşehir Memorial Hospital, Istanbul, Turkey; 5 Department of Biostatistics, Acıbadem Mehmet Ali Aydınlar University, Istanbul, Turkey; 6 American Robert College of Istanbul, Istanbul, Turkey; University of Bern, University Hospital Bern, SWITZERLAND

## Abstract

To practically determine the effect of chloride (Cl) on the acid-base status, four approaches are currently used: accepted ranges of serum Cl values; Cl corrections; the serum Cl/Na ratio; and the serum Na-Cl difference. However, these approaches are governed by different concepts. Our aim is to investigate which approach to the evaluation of the effect of Cl is the best. In this retrospective cohort study, 2529 critically ill patients who were admitted to the tertiary care unit between 2011 and 2018 were retrospectively evaluated. The effects of Cl on the acid-base status according to each evaluative approach were validated by the standard base excess (SBE) and apparent strong ion difference (SID_a_). To clearly demonstrate only the effects of Cl on the acid-base status, a subgroup that included patients with normal lactate, albumin and SIG values was created. To compare approaches, kappa and a linear regression model for all patients and Bland-Altman test for a subgroup were used. In both the entire cohort and the subgroup, correlations among BE_Cl_, SID_a_ and SBE were stronger than those for other approaches (r = 0.94 r = 0.98 and r = 0.96 respectively). Only BECl had acceptable limits of agreement with SBE in the subgroup (bias: 0.5 mmol L-1) In the linear regression model, only BE_Cl_ in all the Cl evaluation approaches was significantly related to the SBE. For the evaluation of the effect of chloride on the acid-base status, BE_Cl_ is a better approach than accepted ranges of serum Cl values, Cl corrections and the Cl/Na ratio.

## Introduction

Chloride (Cl) is the major extracellular strong anion, and there is no suspicion that hyperchloremia and hypochloremia result in metabolic acidosis and alkalosis, respectively [1]. However, the definitions of normochloremia, hypochloremia and hyperchloremia are unclear. Currently, four approaches are practically used in different settings to detect the effect of Cl on the acid-base status: 1) the use of accepted limits of serum Cl values; 2) chloride corrections, such as Cl deficiency/excess (Cl_def/exc_) and Cl modification (Cl_mod_); 3) the serum sodium (Na)-Cl difference, such as base-excess chloride (BE_Cl_); and 4) the serum Cl/Na ratio [1–5]. Each of these approaches has different fundamental concepts. In traditional approach, dischloremia is defined in accordance with limits for observed Cl (Cl_obs_), however, different normal limits for Cl_obs_ such as 98–106 mmol L^-1^, 100–108 mmol L^-1^ and 95–110 mmol L^-1^ are accepted in literature [6–8]. On the other hand, chloride corrections claim that Cl_obs_ levels should be corrected in accordance with Na changes [2, 3]. But, in Cl_def/exc_ and Cl_mod_, since accepted normal values for Na and corrected Cl (Cl_corr_) are different, it can be reached to the different results. Cl/Na ratio is the new approach and there are different accepted normal limits for it in literature [5, 9, 10]. As for the BE_Cl_, it is the only consistent parameter with the Stewart Approach because apparent strong ion difference (SID_a_), which is used to evaluate electrolyte effect in Stewart Approach, also bases on the difference between Na and Cl [4]. For these discrepancies, for a patient, different Cl effects on the acid-base status can be calculated by using each of them. These different results may lead to different diagnoses and treatments. Hence, the important question is which approach is the most accurate. Therefore, the aim of this study is to investigate which approach to the evaluation of the effect of Cl is the most reliable.

## Materials and methods

### Study population

**Fig 1:**
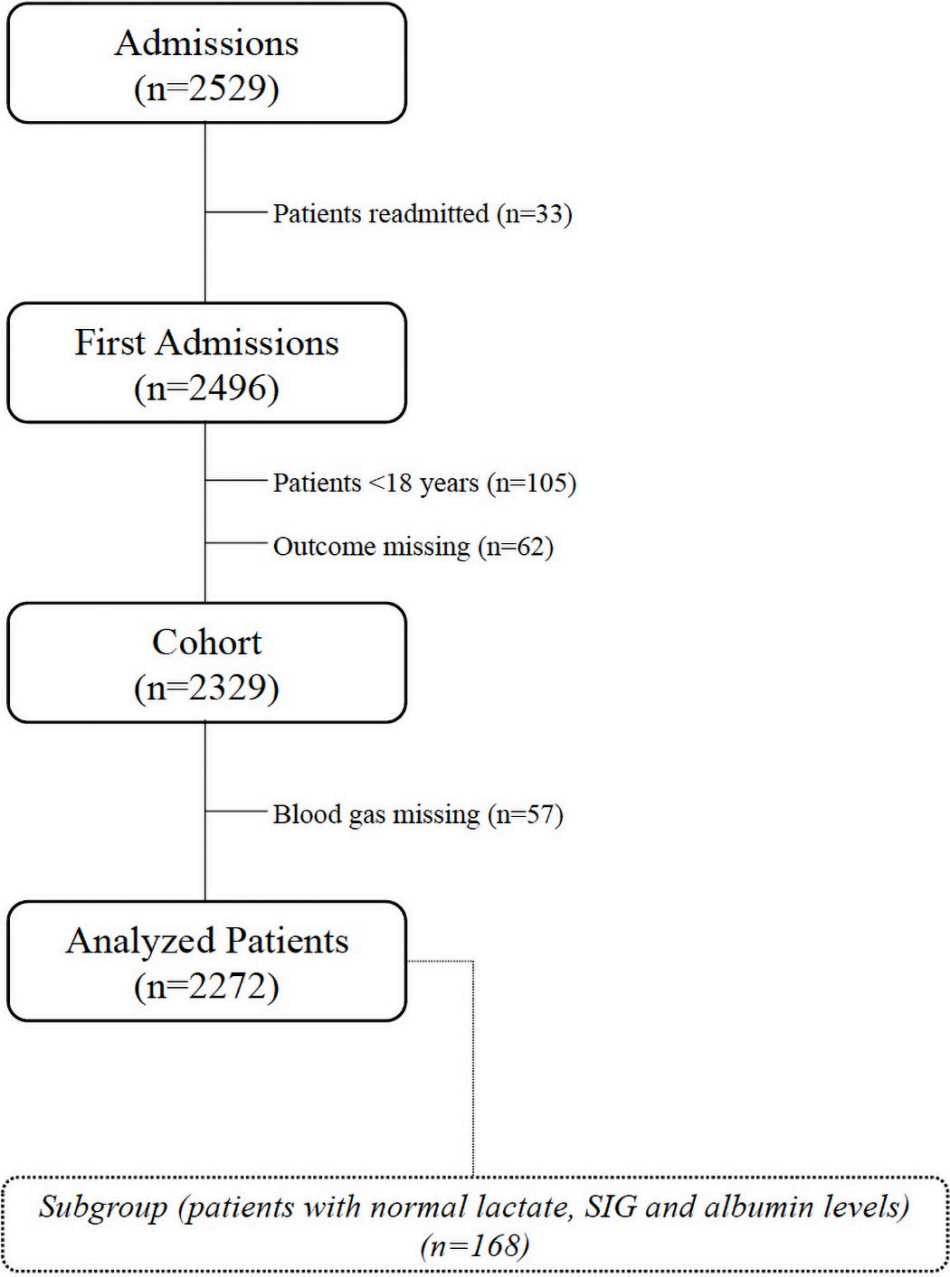
Study flowchart.

This study was approved by the Acıbadem University Medical Research Assessment Council (ATADEK, 2016-18/1) on 10.11.2016. All data were fully anonymized without restriction after the ethics committee approval and ethics committee waived the requirement for informed consent. Data collection was started on 01.01.2018 and 2529 patients who were admitted to the tertiary intensive care unit (ICU) in Acıbadem International Hospital between January 1^st^, 2011, and January 1^st^, 2018 were retrospectively evaluated. Inclusion criteria were age>18 years, arterial blood gas samples and albumin levels at the ICU admission. Exclusion criteria were readmissions and missing outcomes (Fig 1).

### Database

Patients demographics, blood gas and laboratory data were collected from the Acıbadem Health Group Database as from 01.01.2018. ABL 800 (Radiometer, Denmark, Copenhagen), which employs an ion-selective electrode, was used for blood gas analysis. At ICU admission, demographic data (age, sex), diagnosis (medical, elective and emergency surgeries), Acute Physiology And Chronic Health Evaluation II (APACHE II) score, pH, PaCO_2_ (mmHg), Na (mmol L^-1^), K (mmol L^-1^), Ca (mmol L^-1^), Cl (mmol L^-1^), HCO_3_ (mmol L^-1^), SBE (mmol L^-1^), SID_a_ (mmol L^-1^), effective strong ion difference (SID_e_) (mmol L^-1^), strong ion gap (SIG) (mmol L^-1^), BE_Cl_ (mmol L^-1^), serum lactate (mmol L^-1^), albumin (g L^-1^) and outcomes were recorded.

## Approaches of chloride evaluation, accepted serum normal values for Na and Cl (Na_n_ and Cl_n_) and calculations

### Traditional approach

The effect of Cl is evaluated in accordance with accepted limits of Cl_obs_. In this study, serum Cl_n_ ranges for Cl_obs_ were defined as 98–106 mmol L^-1^ [6]. Cl_obs_ levels which are below 98 mmol L^-1^ were defined as hypochloremia whereas Cl_obs_ levels which are above 106 mmol L^-1^ were defined as hyperchloremia.

### Chloride corrections

In this concept, it is claimed that Cl_obs_ should be corrected in accordance with serum Na changes. There are two approaches in literature;

1. **Cl**_**def/exc**_
  For the Cl_def/exc_, accepted serum Na_n_ and Cl_n_ were 142 mmol L^-1^ and 104–108 mmol L^-1^ respectively [2] Firstly, Cl_corr_ is calculated as below;
  Cl_corr_ = (Cl_obs_ x (Na_n_ / Na_obs_))
  If calculated Cl_corr_ levels are between 104–108 mmol L^-1^, Cl_def/exc_ is equal to zero and it indicates normochloremia.
  If calculated Cl_corr_ levels are below 104 mmol L^-1^, Cldef/exc is calculated as below; Cl_def/exc_ = (104 mmol L^-1^—Cl_corr_)
  Since Cl_def/exc_ will have positive value, it will indicate the alkalotic effect due to hypochloremia.
  If calculated Cl_corr_ levels are above 108 mmol L^-1^, Cl_def/exc_ is calculated as below; Cl_def/exc_ = (108 mmol L^-1^—Cl_corr_)
  Since Cl_def/exc_ will have negative value, it will indicate the acidotic effect due to hyperchloremia.
2. **Cl**_**mod**_
  For the Cl_mod_, accepted serum Na_n_ and Cl_n_ were 140 mmol L^-1^ and 102 mmol L^-1^ respectively [3]
  Cl_corr_ is also calculated as the same equation as below;
  Cl_corr_ = (Cl_obs_ x (Na_n_ / Na_obs_))
  However, if the calculated Cl_corr_ level is 102 mmol L^-1^, Cl_mod_ is equal to zero and it indicates normochloremia.
  If calculated Cl_corr_ levels are below 102 mmol L^-1^, Cl_mod_ is calculated as below;
  Cl_mod_ = (102 mmol L^-1^—Cl_corr_)
  Since Cl_mod_ will have positive value, it will indicate the alkalotic effect due to hypochloremia.
  If calculated Cl_corr_ levels are above 102 mmol L^-1^, Cl_mod_ is calculated with the same formula as below;
  Cl_mod_ = (102 mmol L^-1^—Cl_corr_)
  Since Cl_mod_ will have negative value, it will indicate the acidotic effect due to hyperchloremia.

### Cl/Na ratio

Normal limits for serum Cl/Na ratio are accepted as between 0.75–0.80 [5]. There are no accepted serum Na_n_ and Cl_n_ values in this concept.

If Cl/Na ratio is between 0.75–0.80, it is defined as normochloremia.

If Cl/Na ratio is below 0.75, it indicates the alkalotic effect due to hypochloremia.

If Cl/Na ratio is above 0.80, it indicates the acidotic effect due to hyperchloremia.

### BE_Cl_

In this concept, the difference between serum Na and Cl should be 32 mmol L^-1^ [4]. BE_Cl_ is calculated as below;

BE_Cl_ = (Na—Cl—32 mmol L^-1^)

If BE_Cl_ level is zero, it indicates normochloremia.

If BE_Cl_ levels are below zero, it indicates the acidotic effect due to hyperchloremia.

If BE_Cl_ levels are above zero, it indicates the alkalotic effect due to hypochloremia.

### Other calculations

SID_a_, SID_e_ and SIG, were calculated as follows: [11]

SID_a_ = ([Na]+[K]+[Ca])—([Cl]+[lactate])

SID_e_ = (2.46x10^-8^x(P_a_CO_2_ / 10^-pH^)) + (Albumin (g L^-1^) x (0.123xpH-0.631))

SIG = SID_a_—SID_e_

### The subgroup

To clearly demonstrate only the effects of Cl on the acid-base status, a subgroup that included patients with normal lactate (≤1.6 mmol L^-1^), albumin (≥35 g L^-1^, the lowest value of normal laboratory limits) and SIG (0–2 mmol L^-1^) values was created (Fig 1) [3, 12].

### Statistical analysis

All data were analysed by using SPSS version 27. The Kolmogorov smirnov test was used to detect normal distributions. The data were presented as percentages or medians and interquartile range (IQR). The effects of chloride on the acid-base status according to the different approaches were validated by the SBE and SID_a_ because they are parameters which indicate the total metabolic effects and the metabolic effects of electrolytes respectively. The agreement between each pair of approaches was analysed with the Kappa test. The Pearson correlation was used for all correlations. According to revised SBE formula [13], whose components are all electrolytes, albumin, lactate, SIG and PaCO2, for all patients, all Cl evaluation parameters (Cl_obs_, Cl_def/exc_, Cl_mod_, BE_Cl_, Cl/Na ratio), Na_obs_, K, Ca, albumin, lactate, SIG and P_a_CO_2_ were added to the multivariate linear regression models to determine their relationship with the SBE. In the subgroup, the Bland & Altman test was used to determine the limits of agreement between SBE and each of Cl_mod_, Cl_def/exc_ and BE_Cl_. Acceptable agreement was a bias of up to ±1 mmol/L and the limits of agreement were less than ±1.96 mmol L^-1^. A p-value <0.05 was considered statistically significant.

## Results and discussion

**Fig 2:**
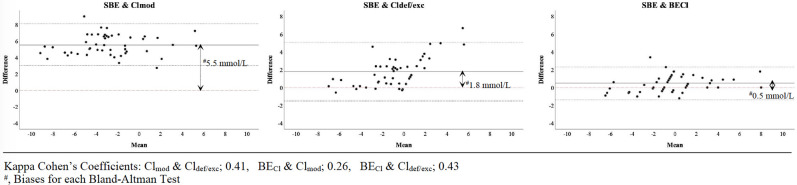
Limits of agreement for chloride approaches in subgroup.

2272 patients were included in this study (Fig 1). According to five chloride evaluation approaches, patients were differently distributed in hypo, normo and hyperchloremia groups (Table 1). The median values of SID_a_, SBE, BE_Cl_, Cl_def/exc_, Cl_mod_ and the Cl/Na ratio were 34.8 (6.8), -1.0 (5.3), -1.0 (6.0), -1.5 (5.0), -6.0 (6.1) and 0.77 (0.04) in all patients, respectively (Table 2). In both the entire cohort and the subgroup, correlations among BE_Cl_, SID_a_ and SBE were stronger than those for other approaches (r = 0.94, r = 0.98 and r = 0.96 respectively) (Table 3). All measures of agreement between the approaches were weak (kappa<0.50) (Table 4). Only BE_Cl_ had acceptable limits of agreement with SBE in the subgroup (bias: 0.5 mmol L^-1^) (Fig 2). In the linear regression models for all patients, P_a_CO_2_, BE_Cl_, K, Ca, lactate, albumin and SIG were significantly related to SBE (p<0.001 for all) whereas Na_obs_, Cl_obs_, Cl/Na ratio, Cl_def/exc_ and Cl_mod_, were not. Additionally, every 1 mmol L^-1^ increase in BE_Cl_ was associated with a 0.90 mmol L^-1^ increase in the SBE (p<0.001) (Table 5).

**Table 1:** Distribution of hypo, hyper and normo-chloremic patients in accordance with different chloride approaches.

		Hypochloremia n (%)	Normochloremia n (%)	Hyperchloremia n (%)	Total n
Observed Chloride	Cl_obs_^6^	196 (8.6)	1063 (46.8)	1013 (44.6)	2272
Chloride corrections	Cl_def/exc_^2^	301 (13.2)	538 (23.7)	1433 (63.1)	2272
	Cl_mod_^3^	263 (11.6)	1 (0.0004)	2008 (88.4)	2272
Cl/Na Ratio	Cl/Na^5^	488 (21.5)	1247 (54.9)	537 (23.6)	2272
Na-Cl difference	BE_Cl_^4^	885 (39.0)	210 (9.2)	1177 (51.8)	2272

BE_Cl_, base excess-chloride; Cl_def/exc_, chloride deficiency-excess; Cl_mod_, chloride modification; Cl_obs_, observed serum chloride level.

**Table 2:** Characteristics and outcomes for all patients and subgroup.

	All patients (n = 2272)	Subgroup (n = 168)
Age, year	62 (30)	61.5 (43)
Male, n, (%)	1279 (56.3)	86 (51.2)
APACHE II,	12 (12)	11 (6)
Diagnosis, n, (%)		
Medical	1346 (59.2)	106 (63.1)
Elective surgery	803 (35.3)	58 (34.5)
Emergency surgery	123 (5.5)	4 (2.4)
pH	7.39 (0.09)	7.40 (0.06)
PaCO2, mmHg	38.6 (10.7)	39.2 (7.2)
HCO3-, mmol L^-1^	23.3 (4.6)	24.1 (3.8)
SIDa, mmol L^-1^	34.8 (6.8)	35.5 (3.2)
SIG, mmol L^-1^	2.2 (5.6)	0.95 (1.1)
SBE, mmol L^-1^	-1.0 (5.3)	-0.1 (2.8)
BECl, mmol L^-1^	-1.0 (6.0)	-1.0 (3.0)
Cldef/exc, mmol L^-1^	-1.5 (5.0)	-1.8 (3.2)
Clmod, mmol L^-1^	-6.0 (6.1)	-6.2 (3.9)
Cl/Na ratio	0.77 (0.04)	0.77 (0.03)
Na, mmol L^-1^	137 (6.0)	136 (4.0)
Cl, mmol L^-1^	106 (7.0)	105.5 (7.0)
K, mmol L^-1^	4.0 (0.5)	4.0 (0.5)
Ca, mmol L^-1^	1.12 (0.14)	1.11 (0.10)
Albumin, g L^-1^	29 (8.0)	38 (2.0)
Lactate-, mmol L^-1^	1.4 (1.5)	0.9 (0.5)
Length of ICU stay, day	2.0 (4.0)	1 (2)
Mortality, n (%)	297 (13.1)	14 (8.3)

APACHE II, Acute Physiology and Chronic Health Evaluation; BE_Cl_, base excess chloride;

Cl_def/exc_, chloride deficiency/excess; Cl_mod_, chloride modification; ICU, intensive care unit;

SBE, standard base excess; SID_a_, apparent strong ion difference; SIG, strong ion gap.

Results were given percentage and median (IQR)

**Table 3:** Correlations in all patients and subgroup (r values).

	In all patients (n = 2272)		In subgroup (n = 168)	
	SID_a_	SBE	SID_a_	SBE
**Cl**_**obs**_	0.56	0.37	0.71	0.69
**Cl**_**def/exc**_	0.90	0.49	0.91	0.90
**Cl**_**mod**_	0.92	0.50	0.95	0.93
**Cl/Na ratio**	0.92	0.50	0.95	0.93
**BE**_**Cl**_	0.94	0.49	0.98	0.96

BE_Cl_, base excess chloride; Cl_def/exc_, chloride deficiency/excess; Cl_mod_, chloride modification; Cl_obs_, observed serum chloride level; SBE, standard base excess; SID_a_, apparent strong ion difference;

**Table 4:** Measures of agreements between chloride approaches, kappa values (standard errors).

	Kappa Coefficients (standard errors)
Clobs & Cldef/exc	0.40 (0.015)
Clobs & Clmod	0.16 (0.010)
Clobs & Cl/Na ratio	0.31 (0.016)
Clobs & BECl	0.20 (0.011)
Cldef/exc & Clmod	0.41 (0.017)
Cldef/exc & Cl/Na ratio	0.31 (0.013)
Cldef/exc & BECl	0.43 (0.013)
Clmod & Cl/Na ratio	0.15 (0.008)
Clmod & BECl	0.26 (0.014)
Cl/Na ratio & BECl	0.37 (0.011)

BE_Cl_, base excess chloride; Cl_def/exc_, chloride deficiency/excess; Cl_mod_, chloride modification;

Cl_obs_, observed serum chloride level. Results were given as kappa coefficients (standard error).

**Table 5:** Multivariate linear regression model for SBE (for all patients).

	Coefficient (CI 95%)	p
Cl/Na ratio	7.0 (-34.5; 48.4)	0.743
Cl_def/exc_, mmol L^-1^	-0.08 (-0.21; 0.04)	0.173
Cl_obs_, mmol L^-1^	-0.06 (-0.13; 0.01)	0.085
P_a_CO_2_ mmHg	-0.08 (-0.09; -0.07)	***<0*.*001***
BE_Cl_, mmol L^-1^	0.90 (0.68; 1.11)	***<0*.*001***
K, mmol L^-1^	0.50 (0.27; 0.65)	***<0*.*001***
Ca, mmol L^-1^	0.60 (0.46; 0.73)	***<0*.*001***
Lactate, mmol L^-1^	-1.23 (-1.28; -1.17)	***<0*.*001***
Albumin, g L^-1^	-0.20 (-0.22; -0.17)	***<0*.*001***
SIG, mmol L^-1^	-0.70 (-0.73; -0.67)	***<0*.*001***

Adjusted R^2^: 0.74 Durbin Watson:1.89

BE_Cl_, base excess-chloride; P_a_CO_2_, arterial carbon dioxide pressure; SIG, strong ion gap.

P_a_CO_2_, Na_obs_, Cl_obs_, Cl/Na ratio, Cl_mod_, Cl_def/exc_, BE_Cl_, K, Ca, Albumin, lactate and SIG were included in the model using the enter method. Na_obs_, Cl_mod_, were excluded by model because of their F values and correlations.

### Definitions of hyperchloremic acidosis and hypochloremic alkalosis

Siggaard-Andersen emphasized that the degree of acidity was related to the amount of H^+^ ions after Arrhenius developed the dissociation theory [14]. Stewart and Kellum also mention that the unlimited source of H^+^ ions is water in the body [11, 15]. Under these circumstances, the amount of H^+^ ions in the plasma should be determined by water dissociation. However, terminologically, the use of hyperchloremia and hypochloremia to define Cl effect on acid-base status appears that only Cl level changes cause water dissociation and vice versa. Yet, the Stewart approach defines 3 independent variables to determine H^+^ ions in the plasma, one of which is SID_a_ [2, 11, 14]. In other words, the difference between strong cations and anions determines whether water will dissociate. Since the major cations and anions are Na and Cl in the plasma, water dissociation should depend on the Na-Cl difference, not only the Cl level. Already, Story et al. showed that hyperchloremic acidosis was a type of SID_a_ acidosis [16, 17]. Even so, Tani et al. had defined their groups in accordance with only Cl_obs_ levels, and the mean SID_a_ value of their normochloremic group was 33.9±3.5 although lactate and Na values were normal in this group [6]. Yet, this option can come true in only hyperchloremia, not normochloremia. When we divided our patients into three groups as such in Tani’s study, we also found the median (IQR) SID_a_ value for 1063 patients who accepted normochloremic was 36.3 (5.5). Hence, we think that using accepted Cl limits to define hyperchloremic acidosis or hypochloremic alkalosis is not adequate. Our study clearly showed that using only accepted Cl limits gave us different results when compared to other approaches. It had the weakest correlations and moreover, Cl_obs_ was not associated with the SBE (Tables 3–5). We believe that the reason for incompatible results in some chloride studies may be the use of the accepted Cl limits [6, 7, 18].

### Chloride corrections and the Cl/Na ratio vs BE_Cl_

The other approaches to evaluating the effect of Cl on the acid-base status are chloride corrections, the Cl/Na ratio and BE_Cl_ [2–5]. Since each of them is supposedly based on the Stewart approach, each should correlate with SID_a_. In this study, we found that there were correlations between SID_a_ and each approach in the whole cohort and the subgroup (Table 3). However, the correlation of BE_Cl_ was stronger than those of Cl_def/exc_, Cl_mod_ and the Cl/Na ratio. Furthermore, there was limited agreement among them, and we obtained different results for a single patient by using each of the approaches at the same time (Tables 1 and 4). We think that there may be several explanations of these results. First, the corrections state that Cl_obs_ should be corrected in accordance with Na because Na and Cl should be similarly diluted or concentrated based on the gain or loss of water [2]. This thesis may be valid in vitro. However, it is known that the distributions of Na and Cl among compartments in the body differ because of the Gibbs-Donnan effect, Hamburger shift, absorption mechanisms in the kidney and small and large intestines and the effects of some drugs, such as furosemide [1, 19–21]. Therefore, the Na and Cl concentrations may not increase or decrease in the same manner in different clinical situations. In fact, even if Na and Cl are similarly diluted or concentrated, the corresponding acidosis or alkalosis cannot be explained because SID will be constant. Furthermore, in the classification by Fencl, it is a paradox to use only Na levels to evaluate water excess or deficit while claiming that the dilution or concentration of Cl and Na are the same [2]. Second, the corrections are actually based on the Cl/Na ratio [2, 3]. The Cl/Na ratio is another usable parameter because it is based on the Na_obs_ and Cl_obs_ levels. Thus, Cl/Na ratio studies have consistent results as well [5, 9, 10]. However, in addition to using the Cl/Na ratio, correction approaches create a fictitious Cl level by using the accepted serum normal value ranges for Cl and Na. Yet, it does not make sense to correct Cl when a Cl_obs_ level exists. The Stewart Approach mentions neither limits for Na and Cl nor needed to correct serum Cl levels in accordance with Na changes [11, 15]. The only rule in this concept is the difference between strong cations and anions, which almost equals the Na-Cl difference defined as BE_Cl_ by O’Dell et al. [4, 11]. For this reason, BE_Cl_ should be the most valuable parameter for the evaluation of the effect of Cl. In this study, we found two more important pieces of evidence supporting the superiority of BE_Cl_: a) the bias between BE_Cl_ and SBE in the subgroup that had normal metabolic values except Cl was less than that of the correction approach (Fig 2), and b) BE_Cl_ was significantly related to the SBE in all patients, whereas the correction and Cl/Na ratio approaches were not (Table 5). In other words, BE_Cl_ was the only parameter which determines Cl effect on SBE.

Additionally, these results compel us to discuss whether there are normal value ranges for Na and Cl or not. Although normal values of Na and Cl for physiologic events in the body exist, it appears that they are irrelevant while evaluating the acid-base status if BE_Cl_ is the most reliable approach to evaluate Cl effect.

## Conclusions

A chloride evaluation approach should conform with the electroneutrality law and, consequently, the Stewart approach. According to our results, the best chloride evaluation approach that meets these conditions is BE_Cl_. Hence, the normal value ranges for Na and Cl should be questioned. We believe that this point of view will change the perspective on acid-base evaluation and fluid management.

## References

[1] Pfortmueller CA, Uehlinger D, von Haehling S, Schefold JC. Serum chloride levels in critical illness-the hidden story. Intensive Care Med Exp. 2018;6: 10. 10.1186/s40635-018-0174-5 29654387 PMC5899079

[2] Fencl V, Jabor A, Kazda A, Figge J. Diagnosis of metabolic acid-base disturbances in critically ill patients. Am J Respir Crit Care Med. 2000;162: 2246–2251. 10.1164/ajrccm.162.6.9904099 11112147

[3] Story DA, Poustie S, Bellomo R. Estimating unmeasured anions in critically ill patients: anion-gap, base-deficit, and strong-ion-gap. Anaesthesia. 2002;57: 1109–1114. 10.1046/j.1365-2044.2002.02782_2.x 12428637

[4] O’Dell E, Tibby SM, Durward A, Aspell J, Murdoch IA. Validation of a method to partition the base deficit in meningococcal sepsis: a retrospective study. Crit Care. 2005;9: R464–70. 10.1186/cc3760 16137362 PMC1269470

[5] Durward A, Skellett S, Mayer A, Taylor D, Tibby SM, Murdoch IA. The value of the chloride: sodium ratio in differentiating the aetiology of metabolic acidosis. Intensive Care Med. 2001;27: 828–835. 10.1007/s001340100915 11430538

[6] Tani M, Morimatsu H, Takatsu F, Morita K. The incidence and prognostic value of hypochloremia in critically ill patients. Scientific World Journal. 2012;2012: 474185. 10.1100/2012/474185 22701359 PMC3373177

[7] Shao M, Li G, Sarvottam K, Wang S, Thongprayoon C, Dong Y, et al. Dyschloremia Is a Risk Factor for the Development of Acute Kidney Injury in Critically Ill Patients. PLoS One. 2016;11: e0160322. 10.1371/journal.pone.0160322 27490461 PMC4974002

[8] McIlroy D, Murphy D, Kasza J, Bhatia D, Wutzlhofer L, Marasco S. Effects of restricting perioperative use of intravenous chloride on kidney injury in patients undergoing cardiac surgery: the LICRA pragmatic controlled clinical trial. Intensive Care Med. 2017;43: 795–806. 10.1007/s00134-017-4772-6 28343236

[9] Nagaoka D, Nassar Junior AP, Maciel AT, Taniguchi LU, Noritomi DT, Azevedo LCP, et al. The use of sodium-chloride difference and chloride-sodium ratio as strong ion difference surrogates in the evaluation of metabolic acidosis in critically ill patients. J Crit Care. 2010;25: 525–531. 10.1016/j.jcrc.2010.02.003 20381294

[10] Atalan HK, Güçyetmez B. The effects of the chloride: sodium ratio on acid-base status and mortality in septic patients. TURKISH JOURNAL OF MEDICAL SCIENCES. 2017. pp. 435–442. 10.3906/sag-1602-100 28425228

[11] Kellum JA, Elbers PWG. Stewart’s Textbook of Acid-Base. Lulu Press, Inc; 2013.

[12] Gunnerson KJ, Saul M, He S, Kellum JA. Lactate versus non-lactate metabolic acidosis: a retrospective outcome evaluation of critically ill patients. Crit Care. 2006;10: R22. 10.1186/cc3987 16507145 PMC1550830

[13] Gomez H, Kellum JA. Understanding Acid Base Disorders. Crit Care Clin. 2015;31: 849–860. 10.1016/j.ccc.2015.06.016 26410149

[14] Siggaard-Andersen O. The Acid-base Status of the Blood. 1964. 13989038

[15] Stewart PA. Modern quantitative acid-base chemistry. Can J Physiol Pharmacol. 1983;61: 1444–1461. 10.1139/y83-207 6423247

[16] Story DA, Morimatsu H, Bellomo R. Hyperchloremic acidosis in the critically ill: one of the strong-ion acidoses? Anesth Analg. 2006;103: 144–8, table of contents. 10.1213/01.ane.0000221449.67354.52 16790643

[17] Funk G-C, Doberer D, Heinze G, Madl C, Holzinger U, Schneeweiss B. Changes of serum chloride and metabolic acid-base state in critical illness. Anaesthesia. 2004;59: 1111–1115. 10.1111/j.1365-2044.2004.03901.x 15479321

[18] Neyra JA, Canepa-Escaro F, Li X, Manllo J, Adams-Huet B, Yee J, et al. Association of Hyperchloremia With Hospital Mortality in Critically Ill Septic Patients. Crit Care Med. 2015;43: 1938–1944. 10.1097/CCM.0000000000001161 26154934 PMC4537691

[19] Yunos NM, Bellomo R, Story D, Kellum J. Bench-to-bedside review: Chloride in critical illness. Crit Care. 2010;14: 226. 10.1186/cc9052 20663180 PMC2945073

[20] Berend K, van Hulsteijn LH, Gans ROB. Chloride: the queen of electrolytes? Eur J Intern Med. 2012;23: 203–211. 10.1016/j.ejim.2011.11.013 22385875

[21] Ho KM, Power BM. Benefits and risks of furosemide in acute kidney injury. Anaesthesia. 2010;65: 283–293. 10.1111/j.1365-2044.2009.06228.x 20085566

